# Uche Amazigo: neglected tropical diseases in the pandemic

**DOI:** 10.2471/BLT.22.030222

**Published:** 2022-02-01

## Abstract

Uche Amazigo talks to Andreia Azevedo Soares about the impact of the pandemic on NTD-related activities and the prospects for regaining momentum on the global NTD agenda.

*Q: How did your passion for public health develop?*

A: When I was a little girl, I used to admire the sanitation officers who came to visit my home. They would make unannounced visits to inspect homes and to promote safe water use, environmental sanitation and personal hygiene and my siblings and I really wanted to be like them! I think the fact that my father also raised us to value helping other people also pushed me in the direction of public health. As regards my formal education, my PhD supervisor in Vienna, Professor Horst Aspöck, was a huge influence. He encouraged me to return to my own country to collect field data and I eventually did field research on intestinal parasites in school children. That was my first exposure to the tremendous health challenges school children faced in my country. I was a lecturer at the University of Nigeria at that time and took trips to the government health centres in villages near the university every Monday to listen and talk to outpatients about hygiene and infections. It was during one of those visits that I met a young pregnant woman by the name of Agnes who was suffering from a debilitating and disfiguring rash caused by onchocerciasis. She made a big impression on me.

With the encouragement of Dr Tore Godal who headed the Special Programme for Research and Training in Tropical Diseases (TDR) at WHO from 1986 to 1998, I followed up on the story of Agnes and her community, learning about the appalling plight of women living with the onchocercal skin disease which included the stigmatization they suffered from their husbands and relatives – pioneering research that was presented at the World Health Assembly. That was the beginning of work I did as a research fellow in the Takemi Program in International Health at Harvard School of Public Health in 1991, establishing the evidentiary basis for community-directed treatment (CDT).

*Q: How easy was it to convince the research community and policy-makers of the value of community-directed NTD treatment?*

A: Not easy at all. My colleagues and I faced significant resistance. Based on the research data APOC and TDR had collected from several countries, we were convinced that CDT-based ivermectin programmes yielded better results than those delivered through health systems, but people did not accept it initially. Indeed, some people considered the idea crazy, especially academics and doctors in certain male-dominated circles.

*Q: Was gender discrimination part of the resistance to your work?*

A: Certainly. I faced quite a lot of gender discrimination in the beginning. Even when I was appointed Scientist at APOC and was later responsible for sustainable drug distribution, I sometimes faced discriminatory remarks at meetings. There was similar resistance to my data regarding the skin disease associated with onchocerciasis which up to that point had been thought of as a disease that solely affected the eyes. Thanks to Dr Godal, the data was thoroughly reviewed in TDR and subsequently validated in several country studies. It was the data from APOC countries that helped me overcome challenges regarding CDT strategy also. In 1998, the very first year we asked communities to distribute ivermectin, treatment coverage rose from around 1.5 million people under health system implemented programmes to 14.1 million people. That helped change attitudes among the hierarchy of doctors who doubted that communities could deliver ivermectin safely. It’s worth pointing out that the commitment and dedication of community drug distributors, who do valuable work on a voluntary basis, has continued even during the pandemic and despite the different challenges faced.

“Resource reallocation comes at a cost.”

*Q: How has the COVID-19 pandemic impacted the delivery of NTD-related services?*

A: Many routine well-structured and effective NTD activities have been affected, largely because of the massive reallocation of resources into supporting the fight against the pandemic. These include NTD monitoring and evaluation activities, active case finding and control, water, sanitation and hygiene awareness and health education. It also didn’t help that health workers running NTD programmes were not given funding to procure personal protective equipment, leaving them exposed to infection. We have also faced huge supply chain disruptions that have impacted the production, shipment and custom clearance of medicines. The main challenges relate to coordination and logistics issues in the field. For example, ivermectin supplies came into Nigeria very late last year because we did not submit our national demand data on time. I hope that 2022 will be better in that respect.

I would also like to point out that while some of the guidance offered by WHO has been helpful, some has not. For example, in April 2020, the Organization recommended that mass drug administration and other NTD activities be suspended to reduce the risk of COVID-19 transmission. That caused considerable disruption. I understand the argument for prioritizing COVID-19 response, but it also needs to be understood that resource reallocation comes at a cost. We saw this during the Ebola crisis, when malaria mortality increased dramatically. Predictive modelling suggests that the diversion of resources and disruption of malaria, tuberculosis and HIV control in low- and middle-income countries could cause even more premature death than COVID-19 itself.

*Q: What is the risk of pandemic-related disruptions impacting progress towards elimination and eradication targets for certain NTDs?*

A: Again, mathematical modelling offers some insights, predicting resurgence of some diseases in high transmission areas, which will almost certainly erode the progress made in the last decade if the NTD community is not vigilant. Trachoma is one example. Modelling suggests that trachoma resurgence risks delaying the 2030 elimination target for the disease by two to three years. This is of course regrettable per se, but it also raises concerns about how those extra years are to be funded.

“NTD drug distributors can play a key role in overcoming [COVID-19] vaccine hesitancy.”

*Q: What can be done to regain momentum?*

A: We need to ensure governments step up in the fight against NTDs, especially in a global economic context where donor partners are withdrawing their support. One example of this is the decision on the part of the UK (United Kingdom of Great Britain and Northern Ireland) government to terminate the £ 200 million pounds sterling [US$ 266 million] Ascend [Accelerating the Sustainable Control and Elimination of Neglected Tropical Diseases] project, which has had a huge impact on the control of onchocerciasis in sub-Saharan Africa. That decision alone risks further weakening the public health infrastructure of Nigeria which was already threatened by the COVID-19 pandemic.

And there are other examples where partners are pulling back just at the time when we need them most. I hope they return, and I take every opportunity to entreat them not to forget us and encourage my peers working in NTD initiatives to make themselves heard. But I also call upon national governments to play their part, including of course governments in Africa. Because the sad truth is that African governments have failed to see the fight against NTD as a best buy in public health. That is why NTD programmes are almost always 80% dependent on donors. So, I'm hoping that will change.

Second, we need to ensure that NTD interventions are brought into the mainstream of primary health care, leveraging the enormous power of communities to ensure optimal adaptation and penetration of health services, so that no one is left behind, even those people living on the margins of the formal health-care system. This is of crucial importance for the long-term sustainability and effectiveness of NTD-related health services, but it can also play an important role in the current crisis, with established NTD networks supporting the pandemic response.

*Q: What are the opportunities for collaboration between NTD and COVID-19 interventions?*

A: Thanks to tremendous community engagement and partner support, the NTD networks have come to represent a significant health system resource over the years with more than 4.8 million community members now trained and serving as community drug distributors in sub-Saharan Africa. And they are ready to be trained in the relevant COVID-19 protocols and interventions, including supporting health workers for vaccination. NTD drug distributors can also play a key role in overcoming vaccine hesitancy, because they are trusted by the communities. It is important to remember that some of the drug distributors have been serving their community for more than a decade, some for 18 years. We were consulted on this issue by the Bill & Melinda Gates Foundation at the beginning of 2021 and encouraged them to support collaboration between the national primary health-care agency, which is responsible for distributing COVID-19 vaccines, and the NTD community networks. I am happy to say that our suggestions were heeded and that state and local government bodies working to deliver the Primary Health Care Package are collaborating with the NTD networks, engaging them in vaccine distribution roll-out in many states.

*Q: What is your main concern going forward?*

A: I worry that many countries in our subregion have yet to demonstrate the political commitment and financial support required to strengthen their health systems in response to this pandemic and the next. Because there will be another pandemic. It is just a matter of time. So, I ask myself if we are making the necessary investments in the institutions and building the health system capacity that will allow us to be more resilient in the future. Are we developing the R&D capacity to develop our own vaccines in the future? Are we rebuilding our depleted health workforce? Are we engaging the people, especially populations in rural communities, in co-creating innovative solutions to address the current and future pandemics? Are we investing in active community engagement? Are we changing our attitudes and focusing on reducing social inequities? These are some of the questions that keep me awake at night. And of course, I worry about maintaining the push to eliminate the NTDs, particularly when partners like the UK turn their backs on the poor in NTD endemic communities. We are making progress on many fronts, but we need their continued support. Just as we need sub-Saharan African governments to increase their funding for NTD elimination programmes.

